# Construction Project Manager’s Emotional Intelligence and Team Effectiveness: The Mediating Role of Team Cohesion and the Moderating Effect of Time

**DOI:** 10.3389/fpsyg.2022.845791

**Published:** 2022-03-03

**Authors:** Qi Zhang, Shengyue Hao

**Affiliations:** School of Economics and Management, Beijing Jiaotong University, Beijing, China

**Keywords:** emotional intelligence, construction project manager, team effectiveness, team process, project management

## Abstract

The emotional intelligence of a construction project manager plays an essential role in project management, and recent developments in teamwork have increased the need to explore better ways to utilize teams and achieve effectiveness in the construction sector. However, research that holds the team-level perspective in emotional intelligence studies is lacking, and the mechanism of the construction project manager’s emotional intelligence on team effectiveness remains unexplored. This knowledge gap is addressed by developing a model that illuminates how construction project manger’s emotional intelligence can affect team effectiveness *via* the mediation of team cohesion and the moderation of project team duration. A questionnaire survey was utilized to gather information from construction project teams across 156 leader-member dyads in the Chinese construction industry. The results reveal that construction project manager’s emotional intelligence is positively related to team effectiveness and the team cohesion mediates this cause and effect. Further, project team duration moderates the relationship between team cohesion and effectiveness. This study offers new insight into how project manager can better lead team members toward desired team outcomes from a team perspective and makes an explorative effort in investigating the “time” role in construction project management.

## Introduction

As the project-based organization increased in importance (Bourouni et al., 2014), so did the reliance on project teams. Most work in the construction industry is now performed and relies on project teams (Braun et al., 2012). The construction project team faces the dynamic, complex, and fluctuating situation of the construction industry (Raiden et al., 2004) and the project’s inherently temporary and goal-oriented nature (Clarke, 2010). As such, construction projects have been perennially considered one of the most troublesome contexts to lead people effectively to improve work output and achieve success (Potter et al., 2018). Recent developments in teamwork have increased the need to explore better ways to utilize teams and achieve effectiveness in the construction sector (Azmy, 2015). In an effective team, leaders and followers develop a process that uses diverse perspectives on problems and criteria for evaluating solutions to make complex and innovative decisions and produce excellent performance, attitudes, and behaviors (Chowdhury, 2005; Mathieu et al., 2008). According to Hoevemeyer (1993), the reasons and needs for a project team to achieve effectiveness are 4-fold: effective project team could improve (a) productivity, (b) service quality, and (c) customer satisfaction; (d) effective project team frees the project manager from day-to-day micro-management so that he or she has more time focusing on other tasks. Therefore, a deep understanding of what factors yield desired effectiveness in the construction project context is now required. However, although the project team has been considered in the team research field, this study noticed a lack of research on project team effectiveness. Some studies focus on the contribution of team effectiveness to construction project performance (e.g., Azmy, 2015); few studies explored the antecedent factors of project team effectiveness.

Since the unwillingness or inability of team members to exert sufficient levels of individual effort, team members sometimes fail to develop the best means of combining their capabilities in a concerted direction to achieve team effectiveness (Zaccaro et al., 2009). Therefore, team leadership is essential for team effectiveness (Zaccaro et al., 2009). As construction project is getting mega, eco-friendly, and intelligent, construction project team management practice and research fields have evolved, the heightened expectations of project managers and their leadership are becoming more apparent (Sundqvist, 2019). To better manage projects and achieve effectiveness, recent studies emphasize a nonverbal leadership, emotional intelligence (Rezvani et al., 2016; Montenegro et al., 2021; Zhu et al., 2021), or the ability to perceive and express emotions, to understand and use them, and to manage them to foster personal growth (Salovey et al., 2003). Turner and Müller (2005) pointed out that emotional intelligence may hold the key to improving project managers’ career success as a leader and project results. Individuals with high emotional intelligence are able to make informed decisions, better cope with environmental demands and pressures, effectively handle conflict, communicate in exciting and assertive ways, and make others feel better in their work environment (Love et al., 2011). For project managers who are constantly confronted with communication issues and complex relationships within the project team, formulating satisfactory solutions is essential. Some prior studies have investigated the relationship between construction project manager’s emotional intelligence and project-level output, such as project performance (e.g., Zhang and Fan, 2013; Zhu et al., 2021) or project success (e.g., Maqbool et al., 2017; Montenegro et al., 2021), but are yet to describe the relationship between leader’s emotional intelligence and project team effectiveness. Therefore, the first key research question in this study is as:

*RQ1*. What is the relationship between construction project manager’s emotional intelligence and team effectiveness?

Further, the relationship between construction project manager’s emotional intelligence and team effectiveness can be complicated and cannot be simply explained by direct effect. The contribution of leadership to an effective team relies on the process or mechanism in which team leaders help members work together and achieve a synergistic threshold where collective effort accomplishes more than the sum of individual abilities or efforts (Zaccaro et al., 2009). Thus, this study aims to uncover the process to provide evidence on how construction project manager’s emotional intelligence affects project team and team members and solve the second question:

*RQ2*. What is the mechanism of construction project manager’s emotional intelligence impacts on team effectiveness?

For studying team effectiveness, McGrath developed the I-P-O (Input-Process-Output) model more than 50 years ago (Mathieu et al., 2008). This article employs the I-P-O model to explore the influence mechanism of emotional intelligence on team effectiveness since it provides a framework for conceptualizing the pivotal role of team process for mediating the conversion of inputs to outcomes (Mathieu et al., 2008). Project management researchers like Müller and Jugdev (2012) also highlighted the need for more team process-related variables to deeply examine the relationship between project manager’s leadership and project outcomes. Following-up on their calls, this study selects team cohesion as the process variable in this study. According to Mathieu et al.’s (2008) review, cohesion is one of the most popular researched process variables. Previous research has suggested that leaders motivate followers by shaping shared affect within teams, for example, team cohesiveness (Pillai and Williams, 2004). Further, scholars have indicated that there is no “one style fits all” solution to leadership issues and that the leadership effectiveness is contextual (Nguyen-Duc et al., 2018). Team development theory suggests that project teams mature and develop as they move from the start of a project toward achieving its goals (Senaratne and Samaraweera, 2015). The leader-member relationship that evolves over time in organizations is essentially a gradual deepening of the degree of social exchange (Teboul and Cole, 2005). Therefore, different stages in team development appear to be differently affected by project manager’s emotional intelligence. To explore the factors of team development into the project manager’s emotional intelligence research, this study incorporated the “time” variable into the research model as a moderator in the relationship between construction project manager’s emotional intelligence and team effectiveness.

Conclusively, based on the Input-Process-Output model and team development theory, this study aims to investigate the mediating role of team cohesion and moderating role of time in the relationship of construction project manager’s emotional intelligence and team effectiveness. To develop and test the hypothesized model, this article adopts a leader-member matching approach in Chinese construction and applies a composite-based structural equation modeling (SEM) method, partial least squares (PLS-SEM; Sarstedt, 2020). The following section reviews relevant literature regarding emotional intelligence and team theory and proposes hypotheses among key constructs; subsequently, a methodology for collecting data, measuring constructs, and testing measurement are displayed. Then, a data analysis is provided. Finally, the study’s discussion, implications, and limitations are presented.

## Theoretical Background and Hypotheses

### Emotional Intelligence

To date, there is no unified operational definition of emotional intelligence. Salovey and Mayer’s (1990) definition (which is often referred to as “ability emotional intelligence”) is skill-based and focuses on cognitive aptitude, similar to a traditional intelligence—IQ, emphasizes the procedure of emotional information (Day and Carroll, 2004; Davis, 2011). Some other scholars considered emotional intelligence in a more mixed perspective. They expanded ability emotional intelligence and argued that this concept includes a set of behaviors, characters, traits, skills, and competencies (Kerr et al., 2013). For example, Bar-On (2006) defined emotional intelligence as a cross-section of interrelated emotional and social competencies, skills, and facilitators. The current paper adopted ability emotional intelligence as the theoretical basis, since the latter view or the “mixed emotional intelligence,” frequently and justifiably, criticized for the lack of theoretical clarity (Hughes and Evans, 2018). Hughes and Evans (2018) argues that when a structure is so broad that it can reasonably accommodate nearly everything, it is so changeable in nature and therefore meaningless. Adopting ability emotional intelligence framework helps this study to establish a relatively purer view of project manager’s emotional intelligence. Salovey et al. (2003) constructed ability emotional intelligence from four dimensions or branches: perceiving emotion (Branch 1), using emotion to facilitate thought (Branch 2), understanding emotion (Branch 3), and managing emotion (Branch 4). Branch 1 concerns the ability to identify emotions in oneself and others. Branch 2 means generating, using, and feeling the emotion as necessary to communicate feelings. Branch 3 refers to the ability to comprehend emotional information. Moreover, Branch 4 is defined as the ability to be open to feelings, to regulate them in oneself and others to promote personal understanding and growth.

Over the past few decades, social-psychological and organizational researchers have focused substantial attention on the role of leader influences on workgroup dynamics and performance (Jeong, 2009). A leader’s emotional intelligence is related to managerial effectiveness (Kerr et al., 2013). Leaders with high emotional intelligence scores could sense employees’ emotional reactions and integrate emotional consideration in their leading behaviors (Hur et al., 2011). Salovey and Mayer (1990) also indicated that the behavioral manifestations of emotionally intelligent individuals include individualized consideration, empathy, and respect. Although research has emphasized the significance of construction project manager’s emotional intelligence for achieving project success (Cacamis and Asmar, 2015; Rezvani et al., 2020), less is known about the mechanism that interferes in the emotional intelligence-team effectiveness relationship. Some existing research explores the mechanism that affects the influence of project managers’ emotional intelligence from a project perspective. For example, Rezvani et al. (2016) explained the contribution of project manager’s emotional intelligence to project success through job satisfaction and trust. Other mediators are represented by leadership-related variables, such as transformational, transactional, and *laissez-faire* leadership (Zhan et al., 2018). Some recent studies integrate the project-level context factors, such as project commitment (Zhu et al., 2021) and stakeholder relationships (Montenegro et al., 2021) into the relationship between construction project manager’s emotional intelligence and project performance. The current study noticed that the above studies tried to explain the influence of leader’s emotional intelligence by their attitudes, behaviors, and leadership, or the contextual factors from team external, while they have tended to ignore the impacts of internal team process and development.

Conclusively, the positive correlation between emotional intelligence and project outcome has been known for a long time (Montenegro et al., 2021), but studies that hold the team-level perspective are less. Considering the call for investigating team process-related mechanisms in project managers’ emotional intelligence research (Müller and Jugdev, 2012; Zhu et al., 2021), this study aims to develop a mechanism that explains the relationship between construction project manager’s emotional intelligence and team effectiveness from a team perspective by team cohesion and time development variables.

### Team Effectiveness and the Input-Process-Output Framework

The team literature defines effectiveness in terms of high team-level performance and the consequences a group has for its members (Cohen et al., 1996; Tannenbaum et al., 1996; Piccoli et al., 2006). Effective construction project teams should be able to produce high-quality output (i.e., accomplish assigned tasks and deliver a completed and well-built project compliant with high quality; Azmy, 2015) and reward team members in terms of satisfaction with team membership and working experience (Mohrman et al., 1995). According to the review of Mathieu et al. (2008), the traditional broad classifications that describe team effectiveness usually include team performance and team members’ affective reactions (e.g., satisfaction, commitment, and viability). Thus, this paper builds project team effectiveness in terms of team performance and satisfaction. Specifically, effective project teams are expected to deliver a timely, high-quality product within budget. They should satisfy team members’ needs, including work experience, pay, respect, and cooperation aspects et al. Team performance and satisfaction are also used to examine team effectiveness in project team context in primary studies, such as Piccoli et al. (2006).

Scholars developed the I-P-O model to explore how teams achieve effectiveness (McGrath, 1964). According to Ilgen et al. (2005), the nature of team performance is how inputs lead to processes that in turn lead to outcomes. This study adopted the I-P-O model for several reasons. First, the origin of the I-P-O and other modified models is to figure out *what* predicts team effectiveness and *why* some groups are more effective than others. The adoption of this model meets the requirement of this study for studying how team leader’s emotional intelligence leads to effectiveness. Second, the project team is a purposive structure and needs to achieve its project goals (Fung, 2014). This study believes that the project team nature accords with the I-P-O model (and advanced I-P-O model) in pursuing excellent results. Moreover, the I-P-O framework has had a powerful influence on empirical research, much of which either explicitly or implicitly invokes the I-P-O model in team research (Ilgen et al., 2005).

**Figure 1:**
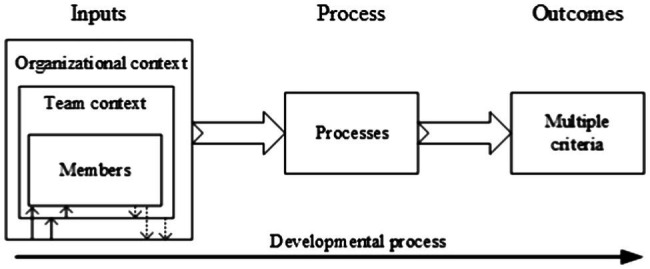
The input-process-output model (Refers to Mathieu et al., 2008). Republished with permission of Sage Publication Inc. Journals from Mathieu et al. (2008); permission conveyed through Copyright Clearance Center, Inc.

In the I-P-O model (Figure 1), *Inputs* describe antecedent factors that enable and constrain members’ interactions. Traditional inputs variables include individual team member characteristics (e.g., competencies and personalities), team-level factors (e.g., task structure and external leader influences), and organizational and contextual factors (e.g., organizational design features and environmental complexity; Mathieu et al., 2008). In this study, the emotionally intelligent leader was set as *Input* since leadership plays its role through shaping members’ interactions (Lyons, 2007) and primary studies, such as Ye et al. (2019) used a similar set in their leadership research.

Many researchers argue that people with higher emotional intelligence can positively impact both team and organization (Rezvani et al., 2016; Maqbool et al., 2017; Zhang et al., 2018). Emotionally intelligent leaders tend to positively experience and express emotion by their emotion regulation ability (Peslak, 2005; Szczygieł and Mikolajczak, 2017). By releasing positive emotion in the workplace, leaders could effectively influence subordinates’ behaviors and attitudes, such as enhancing subordinates’ engagement performance and organizational citizenship behavior (Goswami et al., 2014). As we know, team members’ engagement and organizational citizenship behavior could further contribute to the performance aspect of team effectiveness (Uddin et al., 2018). In addition, positive emotions are likely to increase project managers’ enthusiasm, enabling them to communicate effectively toward their team members (Carmeli, 2003) and be more willing to exchange emotions with subordinates (Cacamis and Asmar, 2015). The positive emotions among every member could motivate team members to express their opinions bravely and promote creative problem solving, thus increasing project team performance (Rezvani et al., 2016). Current results suggest that conflict communication in the project team can negatively affect team outcomes, such as performance and satisfaction (Henderson et al., 2016). It could be further believed that constructional project manager’s emotional intelligence could improve team effectiveness by solving the communication conflict in the project team. On the other hand, according to Salovey and Mayer (1990), behavioral manifestations of emotionally intelligent people are highly transformational; they include demonstrations of care and support, individualized consideration, empathy, and respect. In the project practice, construction project managers need to deal with team members with different personalities and capabilities. Construction project managers with high emotional intelligence have fewer cognitive obstacles when dealing with problems, are easier to understand, and maintain team relationships (Rezvani et al., 2018). By implementing transformational-natural behaviors, project managers inspire team members to keep common goals, create a collaborative atmosphere, and achieve effectiveness (Iqbal et al., 2019). Therefore, this study proposes the following hypothesis:

*Hypothesis* 1: Construction project manager’s emotional intelligence is positively associated with project team effectiveness.

### Team Cohesion

Existing research on team cohesion in the project management field refers to the general definition of team cohesion in organizational context (Fung, 2014; Picazo et al., 2015). Team cohesion describes team members’ commitment to the team’s overall task (Goodman et al., 1987) and the closeness of the interpersonal bonds between team members (Cook and Hunsaker, 1997). Team *process* describes team members’ interactions directed toward task accomplishment and describes how team inputs are transformed to achieve effectiveness (Mathieu et al., 2008). Cohesion is an important process variable (Ledoux et al., 2012) and has always been regarded as one crucial intangible factor for connecting team members and improving team outcomes (Balkundi and Harrison, 2006). As Ilgen et al.’s (2005) argument, cohesion even “goes beyond trust and reflects a strong sense of rapport and a desire to stay together, perhaps extending beyond the current task context.” Besides, team cohesion deals with the strength of the member’s emotional and affective attachment to the larger collective (Bishop and Scott, 2000; Kristof-Brown et al., 2002). Therefore, this study believes that the emotional and affective aspects of team cohesion are valuable to study as the consequence of the project manager’s emotional intelligence.

According to Falahat et al. (2014), emotionally intelligent leaders can subtly influence employees’ practices at work, such as building team cohesion. Quoidbach and Hansenne (2009) also demonstrated that emotional intelligence could constitute an interesting new way of building cohesive teams. As we know, emotionally intelligent project managers tend to express emotion positively (Rezvani et al., 2018). The positive emotion expressed by a leader is usually a signal, which means that the leader is satisfied with existing task performance. This positive signal can make team members feel comfortable and satisfied with the existing communication and cooperation process in work and enhance team cohesion (Chan and Mallett, 2011). The project manager’s emotional understanding and managing ability also contribute to resolving constructive conflict, which was approved as the antecedent variable of team cohesion (Abraham, 2005). From the cultural perspective, some research supports that leaders in institutional collectivism culture tend to use their emotional intelligence to build a collective identity within the team to cultivate the team’s loyalty and cohesion (Miao et al., 2016). China, influenced by Confucian culture, is a significant collectivist nation. Empirically, Rich et al. (2016) found a positive correlation between leaders’ ability emotional intelligence and team cohesion based on the survey conducted in British government departments; Moore and Mamiseishvili (2012) found that the overall score of leader’s emotional intelligence in commercial project teams was significantly positively correlated with team cohesion. Therefore, this paper hypothesizes the following.

*Hypothesis* 2: Construction project manager’s emotional intelligence is positively associated with team cohesion.

Theorists have generally supported a positive effect of team cohesion on team effectiveness. Cohesion reflects team members’ affection and attitude toward the team’s task. Hoegl and Gemuenden (2001) argued that team members with a sense of belonging and togetherness are more likely to engage in collaboration and teamwork. Team cohesion can create a bond among the team, facilitating the uniformity of members and team coordination in task operation and resulting in greater effectiveness (Beal et al., 2003; Lee and Ko, 2019). On the other hand, highly cohesive team members are more willing to exchange work-related experience and value with each other (Lee and Ko, 2019). A team with solid cohesion exerts stronger effort in more efficient planning and may arguably develop more appropriate performance strategies (Gupta et al., 2010). Statistically, studies, such as Fung (2014) and Philip et al. (2013), have indicated that team cohesiveness significantly influences team effectiveness. Therefore, this study posits that as:

*Hypothesis* 3: Construction project team cohesion is positively associated with team effectiveness.

Thus, emotional intelligence helps the construction project managers to improve team cohesion which further leads to team effectiveness. Therefore, team cohesion may mediate the relationships between construction project manager’s emotional intelligence and team effectiveness. The hypothesis is presented as follows.

*Hypothesis* 4: Team cohesion mediates the relationship between the construction project manager’s emotional intelligence and project team effectiveness.

### Team Development Process and Time Variable

Theorists have emphasized that time plays a critical role in team functioning. The time variable in the current paper is the duration of the project team. It could be seen as a continuous variable starting on the first day of a project team.

Conceptually, team researchers have converged on a view of teams as complex, adaptive, and dynamic systems (McGrath et al., 2000). Considering the traditional I-P-O model (McGrath, 1964) fails to capture the complex, adaptive, and dynamic nature of project team, this study employed team development theory and an advanced I-P-O model (Ilgen et al., 2005). Team development theory implies that project teams mature and develop from the beginning of the team forming toward achieving goals (Senaratne and Samaraweera, 2015). The power of the team development process transforms a loose team into an effective group (Sheard and Kakabadse, 2002). Considering the scholars use the *developmental model* (one type of advanced IPO model) to depict the influence of time in the team. Ilgen et al. (2005) indicates that team exists in context as it performs across time. This advanced model reflects how the member interactions are affected differently by how teams qualitatively change over time (Kozlowski et al., 1999). The solid line runs at the bottom of Figure 1 shows the developmental processes unfold over time as teams mature. Existing theory suggests that team processes are likely to be influenced by their progress over time (Mathieu et al., 2008).

According to time development theory, project managers need time to be familiar with team members and implement team-building strategies (Nicolini, 2002). Like ordinary teams, the construction project team could development process is generally along the lines of Tuckman’s (1965) four stages model: forming stage, storming stage, norming stage, and performing stage. In the early stages of team development, construction project managers pay more attention to clarifying the team goal and communicating with external stakeholders (Senaratne and Samaraweera, 2015). This is the exploration stage in the relationship of the leader-member dyad (Teboul and Cole, 2005), and the influence of construction project manager’s emotional intelligence on team cohesion may be limited since developing relationships between followers and leaders is time-consuming activity (Jordan and Troth, 2011). As the team matures over time, the relationship between the project manager and team members develops deeper. Under the influence of reciprocal altruism, team members are more likely to accept the influence from leaders as the leader-member exchange gets deepened (Teboul and Cole, 2005). Besides, considering the external influence from the clients on project managers gets reduced after forming stage, construction project managers could focus more on solving interpersonal conflicts that may occur among members (Senaratne and Samaraweera, 2015), where emotional intelligence may play a better role in improving team cohesion. However, existing empirical studies that focus on the potential moderating effect on the construction project manager’s emotional intelligence and team effectiveness relationship are lacking. Therefore, this article regards this hypothesis as a theoretical exploration and proposes:

*Hypothesis* 5: Project team duration moderates the relationship between the construction project manager’s emotional intelligence and project team cohesion.

**Figure 2:**
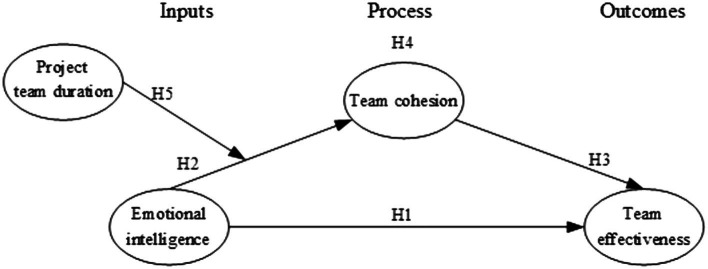
The hypothesized model in the input-process-output framework.

Conclusively, Figure 2 shows the research model of this study.

## Research Methodology

### Research Procedure and Data Collection

This study was conducted in Chinese construction project teams, and a questionnaire survey method was employed. This paper adopted a non-probability sampling method, and Wu et al. (2015) argued that this sampling method is effective for higher response rates and more appropriate for studies in the construction industry. The target population was construction project participants with different levels of work experience, but the sample frames were a subset of the target population, namely, the project manager group and the team member group. The primary targets in both samples were related through common projects, but their roles and responsibilities on these projects are different, i.e., leader and follower.

Considering the COVID-19 pandemic, the author distributed the questionnaires *via* social networks. In collecting the data, a survey kit including an outline of research purpose and a self-reported questionnaire was first sent to construction project managers by personal contacts. Then, through their referrals, the author invited one team member in their team to join the survey. The project manager and team member groups were reminded separately for each leader-member dyad to ensure they answered the questionnaire based on their experience in the same project. As such, the responses of one project manager and one team member constitute one complete team-level questionnaire.

After 2 month recruiting, the survey was distributed to 165 project teams, and a total of 156 leader-member dyads participated in the study, which implies a response rate of 94.54%. For applying the SEM method, most scholars suggest that the number of valid questionnaires should reach 5–10 times latent variables (Bentler and Chou, 1987), and this study meets the requirement. Table 1 outlines the demographic information of the participated project managers. More than 60% of respondents were aged 25–40, showing a rejuvenation of project managers. Among the total number of respondents, 98.08% were male, and 1.92% were female, reflecting the male dominance of the construction industry in China, and this finding is similar to Kim’s (2015). Most of them have Bachelor’s Degree (60.25%) or above (32.69%). More than three-quarters of the participated construction project managers come from state-owned companies (76.92%), reflecting the dominance of state-owned enterprises in the Chinese construction industry (Wang et al., 2006). The average tenure of the participated construction project managers is 11.7 years. They have an average of 4.25 years of work experience as project managers.

**Table 1:** Sample demographics of participated project managers.

Items	Category	Frequency	Percentage
Age	25–30	32	20.51
	31–40	77	49.36
	41–50	40	25.64
	>50	7	7.69
Level of education	Below bachelor	11	7.05
	Bachelor	94	60.25
	Above bachelor	51	32.69
Gender	Male	153	98.08
	Female	3	1.92
Company type	State-owned	120	76.92
	Private	36	23.08
**Mean (years)**			
Job tenure	11.7		
Experience as project manager	4.25		

### Measures

The research hypotheses need to measure four variables: emotional intelligence, team cohesion, project team effectiveness, and project team duration. The Wong and Law Emotional Intelligence Scale (WLEIS) measured the *emotional intelligence* construct, developed for the Chinese context based on the ability model (Wong and Law, 2002). The WLEIS is mainly carried out from four dimensions: self-emotional appraisal, others’ emotional appraisal, use of emotions, and regulation of emotions. Many primary studies conducted in the Chinese project context adopted this scale since it is appropriate to apply to the Chinese context (Law et al., 2008), such as Zhu et al. (2021). Although both self-report and performance tests are widely applied in existing emotional intelligence research, this study adopted the self-reported WLEIS measurement since some scholars argued that the best measurement of emotions and emotional intelligence is self-report because emotional experience is a private internal process. It is not easy for others to observe and judge based on outwardly superficial performance (Connolly et al., 2007). The *team cohesion* construct was measured by the six-item scale developed by Dobbins and Zaccaro (1986). This scale was proved to have high reliability and validity in the project context (Webber, 2008). The *team effectiveness* construct was measured by Li’s (2013) scale developed for the Chinese construction project team. This paper adopted this scale for its appropriateness for target participants. Li’s (2013) scale measures team effectiveness from team performance and team satisfaction. The team performance dimension contains seven items, such as “The project team can complete the project task on schedule” and “Project team cost control within a reasonable range.” The satisfaction dimension contains eight items, such as “In the project team, I am satisfied with the results of my work” and “In the project team, my work was respected by my colleagues and peers.” A five-point Likert scale measured the above three constructs, ranging from 1 (strongly disagree) to 5 (strongly agree). The information of *project team duration* was collected as a single question: (Please recall a project you are practicing on and answer) How long is the project’s duration (accurate to months, e.g., 1 year and six; 5 months). The author manually converted the information to a number in years, e.g., converting “1 year and 6 months” to 1.5 years.

The original survey instrument derived from Wong and Law (2002), Dobbins and Zaccaro (1986) was written in English and then back-translated into Chinese to ensure the equivalence of the original and targeted versions. Three academic experts were consulted to verify the applicability of the questionnaire within the context of Chinese construction projects.

### Common Method Variance

Following the suggestions by Podsakoff et al. (2012), procedural and statistical methods were used to control the common method variance. For process control, the dependent and independent variables in this study were from different sources, which is an obvious way to control common method variance. The project manager group was invited to fill in the emotional intelligence scale and the information of project team duration, and the team member group was invited to fill in the team cohesion and team effectiveness scale. Therefore, the predictors (emotional intelligence and team duration) come from the construction project manager group, while the criterion variables come from the team member group. Besides, the author emphasized that the survey was completely anonymous and that the responses would be confidential. The author also underlined that there were no right or wrong answers. In terms of statistical control, Harman single factor tests were performed on all items of the measured variables. Some researchers argued that this is a less sensitive test and may not detect the presence of common method variance, but more recent research indicates it is a quite meaningful method (Babin et al., 2016; Fuller et al., 2016). The result shows that the explanatory power of the first common factor is 22.08%, thus not reaching the critical point of 40% (Harman, 1960). The result indicates that no single factor can explain most variation, and the common method variance is not a problem for the current study.

### Data Analysis

The data analysis was undertaken in two ways: Confirmatory Factor Analysis (CFA) and PLS-SEM (Partial Least Squares Structural Equation Modeling). The CFA is used to test the measurement model, while the PLS-SEM is used to investigate the relationships among construction project manager’s emotional intelligence, team cohesion, and team effectiveness. PLS-SEM is a causal modeling approach to maximize the explained variance of the dependent latent constructs (Hair et al., 2011). This method is widely used in social science since it is a powerful analytical tool for small sample sizes, formatively measured constructs, and complex models (e.g., mediation, moderation, and moderated mediation; Sosik et al., 2009; Hair et al., 2011). According to Hair et al. (2017), small sample size and formative indicators are two of the top three reasons for PLS-SEM usage. The collected sample sizes are not large (as mentioned above) in the current study, while the hypothesis model is complex, including the mediator and moderator. Further, the emotional intelligence and team effectiveness in this paper are formative constructs. Therefore, this study adopted a PLS-SEM method instead of the traditional SEM method. The current paper employed the Smart-PLS 3.3.5 to conduct the PLS-SEM analysis, and this software is proved to meet the requirements of this study (Joseph et al., 2013).

## Results

### Measurement Model

Reliability and validity were checked for testing the measurement model. Reliability refers to the consistency of a measure. This article used Cronbach’s α to estimate the reliability of each construct. Emotional Intelligence (EI) is comprised of four sub-scales: Self-Emotions Appraisal (SEA), Others-Emotions Appraisal (OEA), Use of Emotion (UE), and Regulation of Emotion (RE). Each of those sub-scales consists out of four sub-constructs. For SEA, the original Cronbach’s α was below the threshold of 0.7, and the outer loading of one item was lower than the threshold of 0.7. After assessing internal consistency reliability and content validity after removal, this item was removed to improve the measurement model. After removing, as shown in Table 2, the Cronbach’s α of all sub-dimensions of EI ranged from 0.708 to 0.794, which are over the threshold of 0.70. The overall Cronbach’s α of the EI construct is 0.704 (>0.7). The team effectiveness (TE) construct includes two sub-constructs: team performance (TP) and team satisfaction (TS). The Cronbach alphas of TP and TS are above the threshold of 0.7 and the overall Cronbach’s α of the TE construct is 0.914 (>0.7). For team cohesion (TC), the Cronbach’s α is also above the threshold of 0.7. Project team duration (PTD) is a continuous variable that measures time and is not required to be consistent. Therefore, the Cronbach’s α of PTD is not presented. The major estimates are demonstrated in Table 2.

**Table 2:** Summary of reliability and validity analysis results.

Construct	Outer loading	Cronbach’s α	CR	AVE
Emotional intelligence (EI)	0.702–0.858	0.704		
Self-emotional appraisal (SEA)		0.708	0.867	0.767
Others’ emotional appraisal (OEA)		0.750	0.815	0.534
Use of emotions (UOE)		0.737	0.817	0.534
Regulation of emotions (ROE)		0.794	0.855	0.598
Team cohesion	0.795–0.830	0.836	0.879	0.551
Team effectiveness	0.764–0.883	0.914		
Team performance		0.892	0.915	0.609
Team satisfaction		0.874	0.901	0.532

Validity refers to the extent to which the scores from a measure represent the underlying variable. The convergent validity was first assessed to evaluate the relative convergence among item measures, reflected by the value of standardized factoring loadings, composite creditability (CR), and average variance extracted (AVE). As reported by Table 2, all item loadings are greater than the benchmark of 0.70. The CR values for the seven sub-constructs ranged from 0.815 to 0.915, satisfying the cutoff of 0.70. The AVE values for each construct ranged from 0.532 to 0.767, larger than the critical value of 0.50. The results confirm a good convergence for all the constructs. The discriminant validity that distinguishes constructs was tested by comparing the square root of a construct’s AVE with the correlative coefficients of other constructs. According to Fornell and Larcker (1981), the square root of the AVE of each construct should be higher than the highest correlation coefficient with any other construct. As shown in Table 3, the square root of the AVE value of each construct exceeds its correlations with all other constructs, meeting the threshold standards and indicating that every construct is truly distinct from other constructs (Fornell and Larcker, 1981). The means, standard deviations (SD), and correlations can also be found in Table 3.

**Table 3:** Descriptive statistics and correlation matrix.

Construct	Mean	SD	1	2	3	4
1.Project team duration	2.43	2.10				
2.Emotional intelligence	4.08	0.26	−0.199^*^	**0.720**		
3.Team cohesion	4.05	0.50	0.240^**^	0.256^**^	**0.742**	
4.Team effectiveness	4.19	0.37	−0.310^**^	0.414^**^	0.592^**^	**0.746**

* *p < 0.05*;

** *p < 0.01*

*In addition, the square root of the AVE is shown on the diagonal highlighted in boldface*.

### Structural Model and Hypothesis Testing

A structural model was run to check the hypotheses and the relationship among the variables, and the results were obtained after bootstrapping by setting samples 5,000 times (Hair et al., 2011). *R^2^* is used to evaluate the path predictive power of the structural model. Generally speaking, 0.25 < *R*^2^ < 0.5 indicates that the model has moderate explanatory power, and *R*^2^ > 0.5 means the explanatory power of the model is high (Ana et al., 2015). The *R*^2^ values of all endogenous variables are greater than 0.25 ( $R_{TC}^{2}$ = 0.280, $R_{\mathrm{TE}}^{2}$ = 0.563), indicating that the model has good explanatory power; see Table 4. The *Q*^2^ value obtained after the Blindfolding test represents the degree to which the path model can predict the initial observation value. As shown in Table 4, the *Q*^2^ values of all two endogenous constructs are considerably greater than zero, providing explicit support for the model’s predictive relevance.

**Table 4:** Explanatory power from *R* square and predictive power from the *Q* square of the endogenous latent variables.

Variables	*R* ^2^	SSO	SSE	*Q*^2^ (=1-SSO/SSE)
Team cohesion	0.280	936.000	812.430	0.132
Team effectiveness	0.563	2340.000	1835.410	0.216

The path coefficient shows the influence of the independent variable on the dependent variable; see Table 5. The final results show that the path coefficient of H1 is 0.397 and the value of *p* is significant at 0.001 level, indicating that construction project manager’ emotional intelligence was significantly positively related to team effectiveness, which supports the H1. The positive relationship between construction project manager’s emotional intelligence and team cohesion (H2) and the positive relationship between team cohesion and team effectiveness (H3) are also approved at the *p* = 0.001 level with the path coefficient at 0.492 and 0.478, respectively. To test the mediating effect (H4), this study applies the procedure suggested by Hair et al. (2017). Partial mediation meets two criteria: (1) emotional intelligence is significantly associated with both team cohesion and team effectiveness and (2) the mediating role of team cohesion in the relationship between construction project manager’s emotional intelligence and team effectiveness is significant, while the effect of emotional intelligence on project performance is significantly reduced. This paper tests the model that does not contain the mediating role of team cohesion and gets the direct path coefficient is 0.636 (*p* < 0.001). After adding the team cohesion as mediating variable, the path coefficient was reduced to 0.397 (*p* < 0.001). Therefore, the H4 was supported with the mediating path coefficient at 0.233, which indicates that construction project manager’s emotional intelligence could improve project team effectiveness by facilitating team cohesion. The moderating role of team duration on the relationship between construction project manager’s emotional intelligence and team cohesion was not significant, with the path coefficient at −0.235 (*p* > 0.05). Therefore, the H5 was not supported.

**Table 5:** Hypotheses testing results.

Hypothesis	Path coefficient	T statistics	Values of *p*	Hypothesis validation
H1 EI → TE	0.397	8.045	^***^	Supported
H2 EI → TC	0.492	6.626	^***^	Supported
H3 TC → TE	0.478	10.886	^***^	Supported
H4 EI → TC → TE	0.233	5.911	^***^	Supported
H5 EI^*^PTD → TC	−0.235	7.504	0.451	Rejected

*EI, emotional intelligence; TC, team cohesion; TE, team effectiveness; and PTD, project team duration*.

*** *p < 0.001*.

## Discussion and Conclusion

### Results Discussion

#### The Effect of Construction Project Manager’s Emotional Intelligence on Team Effectiveness

The positive direct relationship between construction project manager’s emotional intelligence and team effectiveness shows consistency with existing studies to some extent, such as Zhu et al. (2021), Maqbool et al. (2017), and Rezvani et al. (2016), which support a positive relationship between construction project manager’s emotional intelligence and project performance. Although the current study holds a team-level perspective instead of the project-level perspective as in former studies, the consistent result is reasonable since effective teams need to meet the performance requirements (Cohen et al., 1996). For a construction project team, the project performance is readily seen as a team-level performance. Compared to existing studies, this team-level research is a further step since it reveals leader’s emotional intelligence not only contributes to the team’s performance aspect but also works as facilitation to achieving team effectiveness.

On the other hand, this paper also found that some scholars seem to hold an uncertain attitude toward construction project manager’s emotional intelligence. For example, Lindebaum and Cassell (2012) found that construction project managers avoided emotional intelligence under the male-dominated culture in the construction industry and concluded that emotions were unnecessary and inappropriate in United Kingdom construction project workplaces. Lindebaum and Jordan (2012) also suggested that project managers’ emotional intelligence had been exaggerated in the project management field. Although this view comes from a minority compared to the mainstream views, it draws our attention since Chinese construction is also a historically male-dominated industry (Zhang and Zhao, 2015), and most employees in the industry are male (Swider, 2016). The Chinese construction industry has some similarities with the UK’s in male-dominated aspect, the findings of this study show some differences. The cultural differences can explain this inconsistency since compared to western culture, guanxi activities are deeply rooted in Chinese culture and play a vital part in Chinese organizations (Zhan et al., 2018). The guanxi culture indicates that everyone in Chinese society is born not as an individual but as some’s son or daughter, brother, or sister (Fan, 2010). In other words, the individual role is attached to his/her social status and position in a specific relationship. Compared to the western society where construction project managers may think avoiding emotion helps them keep professional (Lindebaum and Cassell, 2012), Chinese construction project managers’ leadership is embedded in their relationships where an individual’s status is defined, i.e., construction project managers refer to *leaders* in the team, and they naturally care about their guanxi with everyone on the team, driven by conventional relationalism. The implementation of guanxi activities was directly influenced by emotions (Barbalet, 2018). The perceiving, using, understanding, and managing emotion abilities are essential that construction project managers naturally use to maintain their status and position in the project team. Therefore, the phenomenon of avoiding emotional intelligence by construction project managers theoretically does not adapt to China’s construction practice, and empirical result approves the contribution of construction project manager’s emotional intelligence. In addition, research time may be another reason that causes the difference. The demonstration of Lindebaum and Cassell’s (2012) research was proposed 10 years ago, and Chinese construction project managers are becoming more familiar with emotional intelligence with the development of society. With the unexpected popularity of the book *Emotional Intelligence* written by Daniel Goleman, the emotional intelligence concept has become popular and has drawn wide attention in Chinese society in recent years. Therefore, although the inconsistency exists, this study treats the finding as supplementary to Lindebaum and Cassell’s (2012) research in a similar male-dominated context and different guanxi cultures.

#### Team Cohesion: The Mediating Role in the Relationship Between Construction Project Manager’s Emotional Intelligence and Team Effectiveness

In terms of the direct influence, the construction project manager’s emotional intelligence was found to be positively related to team cohesion. This result supported the same findings of studies in different contexts, such as Rich et al. (2016), Moore and Mamiseishvili (2012), and Fung (2014), and verified the crucial role of leader’s emotional intelligence in promoting team process in the construction project context. As mentioned before, emotionally intelligent leaders usually express emotions positively (Rezvani et al., 2018). The emotion contagion process from leader to followers can keep the team members excited toward the team vision and be cohesive (Boyatzis and Soler, 2012). Therefore, the author believes that our findings extend existing research to the construction project management field. The positive influence of team cohesion on team effectiveness is also approved, and this result is in line with the prevailing view (Philip et al., 2013). Compared to an ordinary organizational team, due to the project’s uniqueness and complexity, the challenging goal gives members an additional stimulus that inspires members a transcendent insistence on the power of the team (Leopoldo et al., 2009). On this basis, team cohesion exists to promote the individual work harder and enhance the construction project team effectiveness. Chiocchio and Essiembre’s (2009) meta-analysis also reveals that project team cohesion yields a stronger relationship with team output than production team and service team. Therefore, our findings verify the adaption of classical team research in the construction project team.

The significance of the mediating role of team cohesion in the construction project manager’s emotional intelligence-team effectiveness relationship is a novel finding. Existing research suggests that team cohesion is a good mediating variable between leadership and team output, such as transformational leadership (Bass et al., 2003). However, studies that set leaders’ emotional intelligence as *Inputs* are still lacking. According to Salovey and Mayer (1990), behavioral manifestations of high emotional intelligence people are highly transformational; they include demonstrations of care and support, individualized consideration, empathy, and respect. Implementing transformational-natural behaviors could help project managers create a collaborative atmosphere and promote team cohesiveness (Iqbal et al., 2019). Therefore, the similar mediating role of team cohesion in the relationship between leaders’ emotional intelligence and team effectiveness and the relationship between transformational leadership and team effectiveness is reasonable.

#### The Moderating Role of Project Team Duration

**Figure 3:**
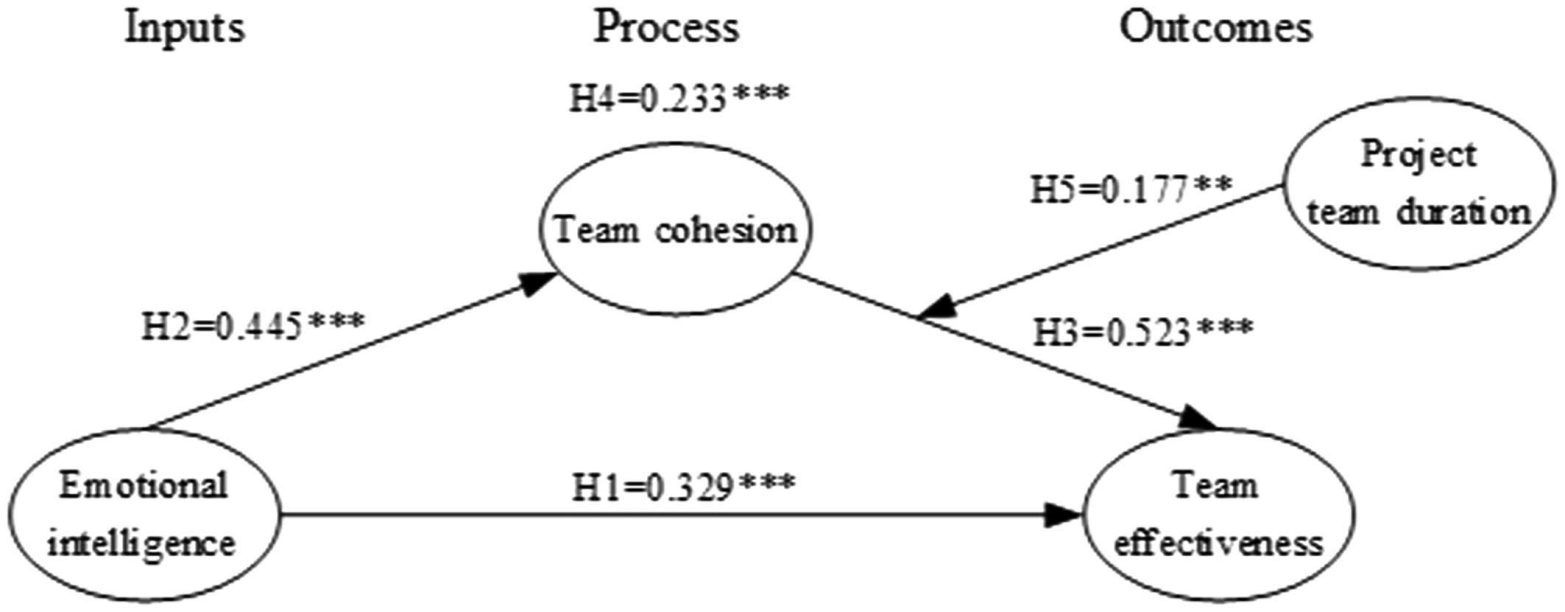
Test results of the final model. ^**^p < 0.01; ^***^p < 0.001.

Result reveals a non-significant negative influence of the team duration on the relationship between construction project manager’s emotional intelligence and team cohesion. This implies that construction project manager’s emotional intelligence on team cohesion remains relatively stable. Considering the influence of team development, this paper tries to modify the hypothesis model. According to the Input-Process-Output model, the improvement of the influence from *input* to *process* originates from the feedback from *process* (team cohesion in the current study) to *input* (construction project manager’s emotional intelligence in the current study; Mathieu et al., 2008), and this feedback is a time-consuming process. This feedback also exists from the *output* (team effectiveness in the current study) to subsequent *process*, further improving the influence from *process* to *output* (Mathieu et al., 2008). Besides, the time development theory suggested that the feedback influence on subsequent *input* would likely be less potent than the influence on *process* (Ilgen et al., 2005). Therefore, it is reasonable to explore the moderating role of team duration on the relationship between team cohesion (process) and team effectiveness (outcome). Using the PLS-SEM, this study found that the positive moderating effect of team duration on the relationship between team cohesion and team effectiveness is significant, with the path coefficient at 0.177 (*p* = 0.006). This result implies that as the project team matures and develops, team cohesion on effectiveness improves. Team theory suggests that when a team enters norming and performing stage, team members accept rules and norms of behavior and relate more deeply to each other and the group’s purpose and task (Tuckman, 1965). Team members in a mature team shift their focus from solving interpersonal relationships to project tasks and achieve flexibility and well-functioning individually (Senaratne and Hapuarachchi, 2009). At this developing stage, team cohesion could help team members keep energy and focus on the task and make consensus decisions. Therefore, team cohesion yields a stronger relationship with team effectiveness when team duration is high. After running the modified model, the path coefficient between variables changed slightly, and the significance of H1-H4 was not influenced. The test results of the modified model are given in Figure 3.

### Theoretically Implications

The current study developed and tested a model based on the Input-Process-Output framework to explore how the construction project manager’s emotional intelligence affects team effectiveness through team cohesion in different team development stages. This study has several implications for the project management field. First, the current study shifts the perspective from the project level to the team level and uses team theory and the Input-Process-Output model to explore the influence of construction project manager’s emotional intelligence. The current study believes that is a novel perspective since although the call for paying attention to the project team has existed for several years (Scott-Young and Samson, 2008; Azmy, 2015), few have investigated the influence of construction project manager’s emotional intelligence at the team level. Researchers have expended much effort exploring a project manager’s impact on a project (Pheng and Chuan, 2006; Davis, 2011; Jalocha et al., 2013) while achieving team effectiveness not only requires good project performance but also need to satisfy team members (Chowdhury, 2005; Mathieu et al., 2008). As academics and practitioners have gradually recognized that the “people” factor plays a crucial role in almost any type of project (Dillon and Taylor, 2015), the need to heighten the role of the team is improving. This study extends existing boundaries for integrating the Input-Process-Output model into leaders’ emotional intelligence research and approves the positive role of construction project managers’ emotional intelligence. In other words, although the influence of construction project manager’s emotional intelligence exists conflict to some extent, as mentioned before, this paper argues that construction project manager’s emotional intelligence works in a positive way, at least at the team level.

Second, this study explored how project cohesion mediates the relationship between construction project manager’s emotional intelligence and team effectiveness. The impact of team cohesion on team outcomes is widely recognized in organizational (Edun, 2015) and project context (Fung, 2014). The current extends the understanding of construction project manager’s emotional intelligence and project team interactions and confirms the impact of team cohesion on project team effectiveness. At the same time, although some studies have explored the mediating mechanism of construction project manager’s emotional intelligence on team output, they paid attention to construction project manager’s behaviors or attitude (Rezvani et al., 2018; Zhang et al., 2018) or external factors, such as stakeholder relationship (Montenegro et al., 2021) or team member’s individual attitude (Zhu et al., 2021), rather than team-level process, such as team cohesion. Therefore, this study answers the calls for exploring variables that potentially mediate the emotional intelligence-project team outcome relationship (Müller and Jugdev, 2012; Azmy, 2015; Zhu et al., 2021) and broaden a new vision to explain the influence of construction project manager’s emotional intelligence on team output.

Thirdly, this research is the first one that provides a more detailed understanding of the role of “time” in the influence process from team cohesion to effectiveness in the construction project team. Although the team development theory has been used in construction project research to explore team interactions (Senaratne and Hapuarachchi, 2009; Senaratne and Samaraweera, 2015), quantitative research is lacking. This research made an effort to combine the construction project team with the existing team theory and elaborated on the different stages of the team process, i.e., from *input* to *process* and from *process* to *output*. The “time” factor plays a different role in different stages. Specifically, project team duration moderates the team cohesion-effectiveness relationship while failing to moderate the construction project manager’s emotional intelligence -team cohesion relationship. This result reveals that the “time” factor may work more effectively in the *process* to *output* procedure than *the input* to *process* procedure. The author believes that this is an explorative result.

### Practice Implications

According to the research results, this paper has several implications for the construction project practice. Firstly, since this study supports a positive relationship between construction project manager’s emotional intelligence and team effectiveness, it is necessary for construction companies and construction project manager groups to pay more attention to construction project manager’s emotional intelligence. Although some scholars argued that it is challenging to improve project performance by training project managers’ emotional intelligence (Lindebaum and Cassell, 2012), this research provides a theoretical basis for Chinese construction enterprises to conduct professional emotional intelligence training, since leader’s emotional intelligence contributes to both performance and team member’s satisfaction aspects. Generally speaking, leadership training in Chinese construction companies has been extensive (Jie, 2019); however, corporate emotional intelligence training that solid, evidence-based guidance for assessing and training emotional skills in organizations remains scarce (Lopes, 2016). Practical emotional intelligence training to improve construction project manager’s emotional intelligence level exerts lasting and long-term effects to project team members. Therefore, this study argues that construction enterprises should establish special emotional intelligence training close to project site management for construction project managers. Secondly, the human resources department could set an emotional intelligence test section for construction project manager recruitment except for emotional intelligence training. Compared to the training, this may be easier when company cost is considered.

On the other hand, the construction project manager group should pay attention to his/her own emotions and emotional intelligence. In the Chinese context, the “emotional intelligence” concept has broad public recognition, often making it far from the academic field and causing difficulties for project managers to recognize the significance of emotional intelligence in leading and management. As such, construction project managers need to enhance their self-awareness of emotional intelligence since this leadership could effectively promote both team process and team output. The ability emotional intelligence school believes that emotional intelligence could be developed with the increase of experience (Davis, 2011), which lays the foundation for construction project managers to increase emotional intelligence level consciously by learning or self-promotion. Besides, team cohesion is a practical path for construction project managers to use emotional intelligence toward achieving effectiveness. As the influence of team cohesion on team effectiveness increase with team development, construction project managers need to pay more attention to the early stage of the project team. The basis of this recommendation is as: as teams mature and develop, team cohesion could affect team effectiveness dependently better than a newly formed team; and if the construction project manager wants to create effectiveness at the beginning of the team, they need to make more effort. As we know, project team members have different characteristics and skills. There exists a tough time for construction project manager to unify the whole team. Our findings suggest that it is better for construction project managers to build team cohesion as soon as possible to help team cohesion works for team effectiveness.

### Limitations and Further Research

Despite its theoretical and practical contributions, this study has certain limitations. First, although this paper explained the reason for choosing the ability emotional intelligence model, considering the difference between ability emotional intelligence and mixed emotional intelligence, there may be different results when the mixed model was adopted. Future research could explore more emotional intelligence frameworks, such as the non-cognitive model (Bar-on, 2006), competency-based model (Goleman, 1998), and the trait model (Petrides and Furnham, 2006), and compare the result with this study. Further, considering the accessibility of team data, this study only collected responses from 156 leader-member dyads. Although the PLS method was adopted for a small sample size, a larger sample is needed for future research. Last but not least, the team theory offers many other team-level process variables for project researchers, such as team confidence, empowerment, climate, collective cognition, and shared mental model (Mathieu et al., 2008). This article argues that more mediating mechanisms could be tested for future research.

## Data Availability Statement

The original contributions presented in the study are included in the article/supplementary material, further inquiries can be directed to the corresponding author.

## Author Contributions

SH designed the research question, guided the research design, and involved the interview. QZ designed the interview process, involved the interview, and drafted the manuscript. All authors coordinated and contributed to the article.

## Funding

This research was funded by the Fundamental Research Funds for the Central Universities (2018YJ053).

## Conflict of Interest

The authors declare that the research was conducted in the absence of any commercial or financial relationships that could be construed as a potential conflict of interest.

## Publisher’s Note

All claims expressed in this article are solely those of the authors and do not necessarily represent those of their affiliated organizations, or those of the publisher, the editors and the reviewers. Any product that may be evaluated in this article, or claim that may be made by its manufacturer, is not guaranteed or endorsed by the publisher.
